# *Trichophyton* as a Rare Cause of Postoperative Wound Infection Resistant to Standard Empiric Antimicrobial Therapy

**DOI:** 10.1155/2018/3483685

**Published:** 2018-12-20

**Authors:** Sheema Gaffar, John K. Birknes, Kenji M. Cunnion

**Affiliations:** ^1^Department of Pediatrics, Eastern Virginia Medical School, 700 West Olney Road, Norfolk, VA 23507, USA; ^2^Division of Pediatric Neurosurgery, Children's Hospital of the King's Daughters, 601 Children's Lane, Norfolk, VA 23507, USA; ^3^Division of Infectious Diseases, Children's Hospital of the King's Daughters, 601 Children's Lane, Norfolk, VA 23507, USA; ^4^Children's Specialty Group, 811 Redgate Avenue, Norfolk, VA 23507, USA

## Abstract

Fungal infections are rare causes of acute surgical wound infections, but *Candida* is not an infrequent etiology in chronic wound infections. *Trichophyton* species is a common cause of tinea capitis but has not been reported as a cause of neurosurgical wound infection. We report a case of *Trichophyton tonsurans* causing a nonhealing surgical wound infection in a 14-year-old male after hemicraniectomy. His wound infection was notable for production of purulent exudate from the wound and lack of clinical improvement despite empiric treatment with multiple broad-spectrum antibiotics targeting typical bacterial causes of wound infection. Multiple wound cultures consistently grew *Trichophyton* fungus, and his wound infection clinically improved rapidly after starting terbinafine and discontinuing antibiotics.

## 1. Introduction

*Trichophyton* fungi are a common cause of tinea capitis but have not been reported as a cause of postsurgical scalp wound infection [1]. Here, we report a 14-year-old male with a chronic wound infection after hemicraniectomy that was eventually determined to be caused by *Trichophyton*.

## 2. Case Report

This case report was reviewed by the local IRB at Eastern Virginia Medical School (18-09-NH-0217) and deemed “not human subjects research.”

**Figure 1:**
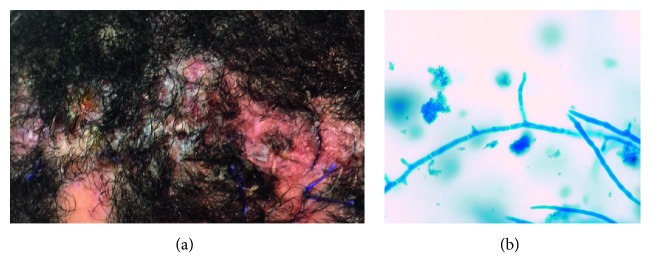
(a) Scalp wound appearance at the time first culture was obtained that grew *Trichophyton*. (b) Lactophenol cotton blue stain of the colony material showing hyphae and budding.

A 14-year-old male with a past medical history of mild intermittent asthma presented in December 2017 with a subdural empyema resulting from direct extension from frontal sinusitis. His intracranial abscess was surgically drained as part of a hemicraniectomy procedure. Cultures of his intracranial abscess grew *Streptococcus intermedius*, and he was treated with antibiotics for 2 months. His craniectomy plate was reimplanted in June 2018. Two and a half weeks later, he presented with pain and mild wound dehiscence. Several patches of alopecia along the edges of the wound were noted. A culture of purulent material expressed from the wound grew rare *Pseudomonas aeruginosa*. Despite appropriate antibiotics, there was no clinical improvement in drainage or pain, leading to surgical removal of the reimplanted bone in July 2018. One week postoperatively, he reported increasing pain along the incision while being treated with ceftazidime. There were fluctuance and profound tenderness to palpation along the incision site (Figure 1(a)), and a new thick purulent discharge was expressible from the wound. There were a few patchy areas of alopecia along the wound edges, which at the time were attributed to preoperative shaving, frequent wound cleaning, and removal of dressings and tape. The skin in the areas of alopecia was not scaly. Expressed purulent drainage was cultured, and his antibiotics were switched to vancomycin and meropenem. The new wound culture grew hyphal fungus on a blood agar plate after 4 days. In total, six wound cultures of the expressed purulent material from the wound were performed over two weeks, and all grew colonies with branched hyphae morphologically consistent with *Trichophyton* (Figure 1(b)). Once identification of *Trichophyton* was made, he was started on oral terbinafine and his antibiotics were discontinued. His wound infection improved rapidly thereafter with decreasing amounts of purulent drainage, fluctuance, pain, and tenderness. After three weeks of terbinafine treatment, all discharge and tenderness had resolved. Follow-up after 6.5 weeks of terbinafine treatment demonstrated an optimal response, including patchy regrowth of hair, and terbinafine was discontinued.

University of Texas Health San Antonio (UTHSA) identified the fungal species as T. tonsurans. Fungal susceptibility testing was also performed by UTHSA. Identification included phenotypic characterization and DNA sequencing of the following targets: ITS, D1/D2, and TUB. This isolate was susceptible to terbinafine (MIC = 0.008 mcg/ml) and griseofulvin (MIC = 1 mcg/ml).

## 3. Discussion

Chronic wounds can be categorized into progressive ulcerative wounds (e.g., diabetic foot ulcers, decubitus ulcers, and venous stasis ulcers), slow healing wounds that require debridement (e.g., burns), and nonhealing incisions [2]. Chronic wound studies typically focus on adult patients. One study of 915 chronic surgical wounds over 4 months reported a 23% incidence of fungal infection [2], while another study of 824 nonchronic surgical wounds reported a 2% incidence [3]. In another survey of polymicrobial chronic wound infections, *Candida albicans* was implicated as the most common contributor [4]. Thus, the risk of fungal wound infection appears to be much greater for chronic as compared to nonchronic wounds. The vast majority of postoperative fungal wound infections are caused by *Candida*. Risk factors for delayed wound healing from *Candida* wound infections included occlusive dressings and treatment with antibacterial ointments [4, 5]. *Aspergillus* has been reported to cause fungal endophthalmitis after ophthalmologic surgery, while *Trichophyton* has been reported to cause fungal keratitis after cataract surgery [6]. Cases of postoperative infection from *Trichophyton* have also been reported after hair transplantation [7].

Tinea capitis caused by *Trichophyton* is common in pediatrics. Clinical manifestations of tinea capitis can be categorized into alopecic and inflammatory [8]. Tinea capitis causing alopecia appears as a few large-diameter lesions, called microsporosis, or as many small alopecic lesions, called trichophytosis [9]. Inflammatory tinea capitis has a similar divergence per wound characteristic. Yellow crusts with associated odor are characteristic of favus reaction, while suppurative exudate and edema with associated pain are characteristic of kerion [9]. The crusting and spongy subcutaneous edema, which is sometimes accompanied by a thick white exudate, in kerion-specific inflammatory tinea capitis results from a T cell-mediated hypersensitivity reaction to *Trichophyton*, rather than subcutaneous infection [10]. The patient we describe had a chronic wound infection at his hemicraniectomy incision site with significant incisional pain, progressive scalp edema with purulent exudate, and wound dehiscence. The symptomatic characteristics are consistent with kerion. However, the consistent growth of *Trichophyton* from the purulent exudate is not typical for kerion and is more suggestive of a true wound infection rather than allergic reaction to superficial tinea capitis.

The lack of clinical response by this wound infection in the face of multiple broad-spectrum antibiotic regimens was worrisome, leading to concern for potentially unrecognized chronic osteomyelitis of the skull, a retained foreign body, or a multidrug resistant organism. The possibility of a fungal wound infection should also be included in this differential diagnosis. For chronic wound infections, the literature consistently recommends obtaining fungal cultures to isolate the agent [9, 11, 12] because fungal culture is known to have higher sensitivity than fungal microscopy [13, 14]. To our knowledge, *Trichophyton* has not been reported as a primary pathogen in surgical wound infections. In this instance, treatment with terbinafine, an optimal antimicrobial to treat *Trichophyton*, led to clinical improvement in a few days. *Trichophyton* appears to be an exceptionally rare [13] but potential cause of chronic fungal wound infection.

## References

[1] Schmidt M. (2017). Boric acid inhibition of *Trichophyton rubrum* growth and conidia formation. *Biological Trace Element Research*.

[2] Dowd S. E., Hanson J. D., Rees E. (2013). Survey of fungi and yeast in polymicrobial infections in chronic wounds. *Journal of Wound Care*.

[3] Kaya D., Aldirmaz Agartan C., Yucel M. (2007). Fungal agents as a cause of surgical wound infections: an overview of host factors. *Wounds*.

[4] Giandoni M. B., Grabski W. J. (1994). Cutaneous candidiasis as a cause of delayed surgical wound healing. *Journal of the American Academy of Dermatology*.

[5] Paskiabi F. A., Bonakdar S., Shokrgozar M. A. (2017). Terbinafine-loaded wound dressing for chronic superficial fungal infections. *Materials Science and Engineering: C*.

[6] Lin C. M., Pao S. I., Chen Y. H., Chen J. T., Lu D. W., Chen C. L. (2014). Fungal endophthalmitis caused by Trichophyton spp. after cataract surgery. *Clinical and Experimental Ophthalmology*.

[7] Colli P., Fellas A., Trueb R. M. (2017). *Staphylococcus Iugdunensis* and *Trichophyton tonsurans* infection in synthetic hair implants. *International Journal of Trichology*.

[8] Veasey J. V., Miguel B. A. F., Mayor S. A. S., Zaitz C., Muramatu L. H., Serrano J. A. (2017). Epidemiological profile of tinea capitis in São Paulo City. *Anais Brasileiros de Dermatologia*.

[9] Veasey J. V., Muzy G. D. S. C. (2018). *Tinea capitis*: correlation of clinical presentations to agents identified in mycological culture. *Anais Brasileiros de Dermatologia*.

[10] Zaraa I., Hawilo A., Aounallah A. (2012). InflammatoryTinea capitis: a 12-year study and a review of the literature. *Mycoses*.

[11] Stankey C. T., Spaulding A. B., Doucette A. (2018). Blood culture and pleural fluid culture yields in pediatric empyema patients. *Pediatric Infectious Disease Journal*.

[12] Sheth S. P., Ilkanich P., Blaise C. (2018). Complicated *fusobacterium* sinusitis: a case report. *Pediatric Infectious Disease Journal*.

[13] Ang C. C., Tay Y. K. (2010). Inflammatory *tinea capitis*: non-healing plaque on the occiput of a 4-year-old child. *Annals, Academy of Medicine, Singapore*.

[14] Higgins E. M., Fuller L. C., Smith C. H. (2000). Guidelines for the management of tinea capitis. *British Journal of Dermatology*.

